# In Healthy Young Men, a Short Exhaustive Exercise Alters the Oxidative Stress Only Slightly, Independent of the Actual Fitness

**DOI:** 10.1155/2016/9107210

**Published:** 2016-02-17

**Authors:** Maya Finkler, Ayala Hochman, Ilya Pinchuk, Dov Lichtenberg

**Affiliations:** ^1^Department of Physiology and Pharmacology, Sackler Faculty of Medicine, Tel Aviv University, 69978 Tel Aviv, Israel; ^2^Department of Biochemistry and Molecular Biology, George S. Wise Faculty of Life Sciences, Tel Aviv University, 69978 Tel Aviv, Israel

## Abstract

The aim of the present study was to evaluate the apparent disagreement regarding the effect of a typical cycling progressive exercise, commonly used to assess VO_2max_, on the kinetics of* ex vivo* copper induced peroxidation of serum lipids. Thirty-two (32) healthy young men, aged 24–30 years, who do not smoke and do not take any food supplements, participated in the study. Blood was withdrawn from each participant at three time points (before the exercise and 5 minutes and one hour after exercise). Copper induced peroxidation of sera made of the blood samples was monitored by spectrophotometry. For comparison, we also assayed TBARS concentration and the activity of oxidation-related enzymes. The physical exercise resulted in a slight and reversible increase of TBARS and slight changes in the activities of the studied antioxidant enzymes and the lag preceding peroxidation did not change substantially. Most altered parameters returned to baseline level one hour after exercise. Notably, the exercise-induced changes in OS did not correlate with the physical fitness of the subjects, as evaluated in this study (VO_2max_ = 30–60 mL/min/kg). We conclude that in healthy young fit men a short exhaustive exercise alters only slightly the OS, independent of the actual physical fitness.

## 1. Introduction

The relationship between exercise and oxidative stress (OS) depends on the mode, intensity, and duration of exercise. Prolonged heavy physical activity resulted in severe muscle damage [1–5] whereas regular moderate training appears beneficial for OS and health, which is commonly attributed to reactive oxygen species- (ROS-) induced increased expression of antioxidants and adaptation of the muscle phenotype [4, 6, 7]. The effects of a single bout of exhaustive exercise are apparently controversial: many studies provided evidence that such an exercise results in severeoxidative damage [3, 4], whereas other investigators reported that a single bout of physical activity results in slight, not always statistically significant elevation of the concentrations of ROS, if at all [5, 8].

Many factors may contribute to these “somewhat confusing” results [9, 10]. These include (i) differences in physical activity (cycling [11–14]; swimming [14, 15]; running [16, 17]; extreme marches [18]; or rowing [19]), (ii) the intensity of the exercise, (iii) the differences in physical fitness (sedentary [11–15, 17] as compared to athletes [11, 17, 18]), and (iv) the ill-defined nature of the term OS [20–22].

OS cannot be defined in terms of a universal criterion [20] and is therefore estimated based on different “OS biomarkers,” including lipid peroxidation products, DNA fragmentation products, oxidized proteins, carbonyl groups, antioxidant enzymes, and low molecular weight antioxidants (LMWA). The OS, as evaluated by different criteria, using different methods, based on the level of different biomarkers in blood collected at different times (which many publications did not specify), might vary over a relatively large range and have a major effect on the evaluation of OS [18, 23]. Hence, evaluation of exercise-induced OS by determination of different biomarkers may yield different results. As an example, a maximal treadmill exercise test resulted in statistically significant increases in the OS but the increase varied over a wide range, from 6.2% in Protein Carbonyls (PC) to 7.8% in Glutathione Peroxidase (GPx) and 27% in Isoprostanes. Notably, there were large between-individual coefficients of variation (240% in PC; 130% in GPx; 243% in Total Antioxidant Capacity (TAC); and 152% in 5-Isoprostanes [24]).

Other methods of evaluating the OS are based on evaluation of the “overall reductive power,” including the redox activity of the blood and the lag preceding rapid peroxidation* ex vivo* [25]. This lag reflects the susceptibility of serum lipids to copper induced peroxidation* ex vivo* and is a reliable measure of the overall oxidative status [5, 11, 12, 17, 24, 26–31]. Acute strenuous physical exercise may be expected to shorten the lag due to promotion in ROS production and/or due to enhanced consumption of the antioxidants. However, the opposite effects cannot be ruled out because the exercise may activate the enzymatic antioxidant system [30, 32].

Our optimized assay of the susceptibility of serum lipids to* ex vivo* copper induced peroxidation [20, 33] can be conducted on unfractionated serum and is therefore an easy and reliable measure of the overall oxidative status. In a previous study [34], we found that a maximal graded treadmill running test had only slight effects on the kinetics of* ex vivo* copper induced peroxidation of the serum lipids. In the present study, we investigated the effect of a typical cycling progressive exercise, commonly used to assess VO_2max_, on the kinetics of copper induced peroxidation and compared these with the results on exercise-induced changes of TBARS concentration and on the activity of oxidation-related enzymes. Compared to the previous study, the group investigated in this study was larger and more homogeneous. Hence, we could investigate the relationship between physical fitness and the “homeostatic level of OS” and the relationship between physical exercise-induced changes in OS and physical fitness.

## 2. Methods

### 2.1. Protocol

Thirty-two (32) healthy men, aged 24–30 years old (as depicted in Table 1), participated in this research. No one of them smokes or takes any nutritional supplements. Their physical fitness, as evaluated in this study, varied over the range of VO_2max_ = 30–60 mL/min/kg with a normal distribution, which means that some of them were moderately trained while some of them were well trained. The participants fasted for 12 hours prior to the test and received a unified breakfast that included two slices of white bread with strawberry jam (with no fruit) and water. The protocol was approved by the ethics committee of Hillel Yaffe hospital.

### 2.2. Evaluation of Fitness

Fitness was evaluated on the basis of a typical progressive exercise to assess VO_2max_ level. Specifically, the subject cycled on an ergometric bicycle. The test started with a 5 min warm-up and lasted 8–15 min. The initial power output was 50 W and the intensity was increased every minute by 20–30 W. The exercise lasted until the earliest of the following events occurred: (i) the subject had to stop due to exhaustion or (ii) the heart rate stopped rising or (iii) the respiratory exchange ratio increased to a level higher than 1.15 (RER > 1.15).

Exhaled gas was collected in a mixing bag and the rate of oxygen uptake and carbon dioxide release was determined every 10 s by an online respiratory gas exchange analyzer. The test was conducted at the same time of day ±1 h, with a room temperature of 24 degrees' Celsius (±1°C).

### 2.3. Blood Samples

Vein blood was taken from each subject three times, namely, before the workout (at rest), 5 minutes after the intense workout, and one hour after the workout (recovery). At each time point, two tubes of vein blood were taken from each subject; one contained no anticoagulants and was used to measure lipid susceptibility to oxidation in the serum. The other tube was used to measure TBARS and to assay the activity of antioxidant enzymes in the hemolysate made in 0.105 molar buffered sodium citrate solution according to Dodge et al. [35].

### 2.4. Evaluation of OS

The levels of the following biomarkers were assayed in the hemolysate as described in previous studies. Malondialdehyde was assessed by measuring thiobarbituric acid reactive substances (TBARS) [36]. The intra-assay coefficient of variation for the TBARS was about 5%. Detection limit was evaluated as 0.5 *μ*M and the lower quantitation limit was 1.5 *μ*M. The activities of the antioxidant enzymes were measured by conventional assays: Catalase (CAT) [37], Glutathione Peroxidase (GPx) [38], Glutathione Reductase (GR) [39], Glutathione S-Transferase (GST) [40], and Glucose-6-Phosphate Dehydrogenase (G6PD) [41].

### 2.5. Susceptibility of Serum Lipids to Peroxidation

The susceptibility of serum lipids to copper induced peroxidation was evaluated on the basis of kinetic monitoring of copper induced peroxidation* ex vivo*, as described by Schnitzer et al. [42]. Briefly, the conjugated hydroperoxides, formed upon peroxidation, absorb UV light at wavelength of 230–250 nm. Continuous monitoring of the time course of absorbance at 245 nm yields more information than other assays of OS. Specifically, rapid peroxidation is typically preceded by a “lag phase” of slow peroxidation followed by a subsequent free radical chain reaction that propagates until all the peroxidizable lipids (essentially polyunsaturated fatty acids, PUFA) become peroxidized [43]. The lag reflects the protection of serum lipids against peroxidation, the initial absorbance OD245_in_ reflects the background scattering and absorption including initial concentration of hydroperoxides in the serum, OD_max_ reflects the concentration of peroxidizable lipids in the serum, and *V*_max_ is the maximal rate of peroxidation.

### 2.6. Statistical Analyses

Possible correlations and trends were assayed according to Pearson's chi-squared test, one-way repeated measures ANOVA and post hoc tests. A statistically significant difference was defined as *P* < 0.05. All the correlations were analyzed in log units.

## 3. Results and Discussion

### 3.1. Exercise-Induced Time Dependent Changes of OS

**Figure 1:**
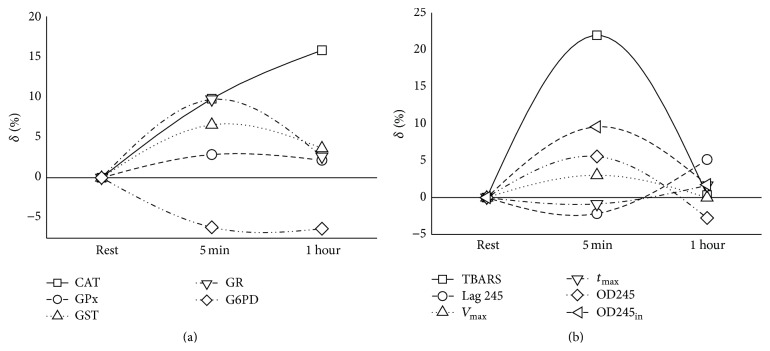
Relative change of biomarkers' values normalized for their values at rest. The relative changes of each of the parameters of Table 2 due to (a) the studied exercise (i.e., the difference between the results obtained before and 5 minutes after exercise) and (b) the postexercise rest (i.e., the difference between measurements obtained 5 minutes after the exercise and at one-hour rest after the exercise).

The average values of the studied OS parameters, as assessed at three different time points, are given in Table 2 and the relative changes of each of these parameters due to the studied exercise and due to the subsequent rest are depicted in Figure 1. As evident from this figure, the studied exercise resulted in small but significant changes in factors associated with the concentration of lipid peroxidation products (TBARS, *V*_max_, OD_max_, and OD245_in_). Significant changes have also been observed in the activity of CAT. G6PD decreased slightly and insignificantly, whereas the activity of all other studied enzymes increased slightly or moderately but the increase did not reach statistical significance and even the statistically significant changes were relatively small (up to ca 20%), as seen in Figure 1. Notably, the concentration of TBARS in the serum increased immediately after the physical exercise, whereas the physical exercise-induced activation of enzymes appeared to be relatively small and, in the case of CAT, it appeared to continue increasing after the physical exercise. We attribute this difference to the different rate of the exercise-induced processes, as described below. Interestingly, the lag preceding rapid lipid peroxidation did not change substantially.

To interpret our results, we first note that the different biomarkers of OS are affected differently by the physical exercise. We attribute these differences to different time dependencies of the concentration of the different biomarkers. Specifically, when physical exercise results in an increase of the concentration or activity of a given biomarker, its level will increase with time and subsequently decrease to its homeostatic level. Both the rate of increase and the rate of the subsequent decrease are different for different biomarkers. Hence, the level of each biomarker changes differently with time, which results in apparently different “levels of OS.”

An obvious example is the difference between estimates of OS based on the activity of different OS-associated enzymes and estimates based on the concentrations of lipid oxidation products or kinetic factors of lipid oxidation [20]. Thus, physical exercise may cause a relatively rapid increase in the concentration of peroxidation products (e.g., TBARS), whereas changes of the activity of OS-associated enzymes are slower and may depend critically in a complex fashion on the time of physical exercise as well as on the time between the end of physical exercise and blood withdrawal [44, 45]. This is particularly important for the studied antioxidative enzymes, which are “erythrocyte enzymes,” namely, their level in the hemolysate reflects formation of new erythrocytes, which is likely to require more time than the duration of the exercise. This is consistent with the higher activity of CAT in the blood withdrawn an hour after the physical exercise than in blood withdrawn 5 minutes after physical exercise, whereas the level of lipid peroxidation products is lower than earlier, probably due to metabolism. Accordingly, the increase of OS due to physical exercise, as evaluated on the basis of the concentration of any biomarker (e.g., MDA) in blood withdrawn at any given time after workout, does not necessarily reflect the full extent of exercise-induced OS. Estimation of the latter factor requires kinetic studies.

Our interpretation of the time courses of* ex vivo* peroxidation can be based on the previous findings that physical exercise induces an increase of the concentrations of lipoprotein lipids, without affecting the tocopherol concentration [1, 2, 13]. The increase in lipid concentrations is likely to result in an increase in the levels of TBARS and of both OD_max_ and *V*_max_. The lack of substantial effect on the lag (Figure 1) accords with the lack of effect of physical exercise on the concentration of tocopherol [13]. Taken together, the latter findings indicate that, in young healthy men, the physical exercise results only in slight changes in the sensitivity of the lipids to peroxidation.

### 3.2. Recovery of “Resting Levels” of OS after Physical Exercise

This work shows that all the studied enzymes except CAT returned to their basic activity one hour after the studied physical activity. These results are similar to those of previous studies that showed that most of the OS criteria that changed “immediately” after workout had returned to their basic values after 1 hour of recovery [12, 15, 16, 51]. As an example, 40 minutes after 800 meters' swimming, both the enzymatic activity of GPx and the concentration of GSH returned to their basic levels, whereas 40 minutes after intense exercise of 100 meters' swimming, none of the biomarkers returned to their basic levels [15]. This probably reflects the difference between aerobic workout (the muscles work for a long time and use only oxygen) and anaerobic workout (the muscles work for a short time without oxygen [14]).

This work indicates that young healthy people can maintain redox homeostasis throughout intense physical exercise. All the enzymes except CAT returned to their basic activity one hour after the studied physical activity. In contrast, CAT's activity increased during the postexercise rest. This trend may either be a result of postexercise continuation of the physical exercise-induced changes and/or of physical exercise-induced long-lasting activation of CAT. The relative contribution of these mechanisms has yet to be determined.

An interesting general observation is that the physical exercise-induced changes in the levels of the various OS biomarkers apparently correlate inversely with the initial level (before the exercise) of the given biomarker (see Supplementary Material available online at http://dx.doi.org/10.1155/2016/9107210). As an example, the dependence of the exercise-induced change in the activity of Catalase on its initial activity was highly significant (*P* < 0.0001) and strong (*R* ^2^ = 0.43). The apparently obvious conclusion is that when the initial OS is high, the physical exercise causes a relatively small increase of the OS and at very high OS, the exercise may even lower it, similar to the results of Theodorou et al. [46]. Unfortunately, this interpretation might be incorrect because the results may be due to the statistical phenomenon commonly designated as “regression toward the mean”; that is, the differences between the consecutive measurements of a given parameter correlate with the basal value [47]. Such an artefactual correlation is not likely to occur between the change in the value of the said parameter and* the average (or sum) of the basal and final values* [47]. This means that if the changes correlate with the mean, the changes are indeed associated with the basal values. We used this protocol and did not find the expected correlation and we can therefore not confirm the above “apparently obvious” interpretation.

### 3.3. Physical Fitness, OS, and Exercise-Induced Changes of OS

All our young and healthy participants were active but their fitness level varied, with a normal distribution, over a relatively large range (VO_2max_ = ca 30 to ca 60 mL/min/kg); that is, some of them were moderately trained; others were well trained. We did not observe a statistically significant correlation between physical fitness and the OS, as evaluated by any of the various methods. This is consistent with several previous reports that found no difference between trained and sedentary subjects [16, 48–50]. In apparent contrast to these results, some researchers found that trained subjects have a lower OS than sedentary subjects [51–54], whereas other researchers found that trained subjects had a higher OS than sedentary subjects [12, 55]. The apparent contradictions between these studies may result from one or more of the following reasons: (i) differences in the methods of testing the fitness, (ii) differences in the method of evaluation of the oxidative stress [22], and/or (iii) the type of physical exercise.

Notably, most of the relevant studies found that the activity of GPx depends on fitness. However, even the dependence of the level of this biomarker on physical fitness varied between different studies. Many articles showed a positive correlation (high GPx activity associated with high fitness) [56, 57]; other studies showed an inverse correlation (GPx activity lower in athletes than in sedentary subjects) [58]. Several other studies showed nonsignificant difference in GPx activity between athletes and sedentary subjects [59].

In relating to the difference in the results due to the way physical fitness was determined, it is worth noting that, in many of the studies that showed no difference or a slight increase in the OS biomarkers, the physical fitness level was based on the measurement of VO_2max_, whereas, in most of the publications that showed a statistically significant correlation, evaluation of the physical fitness was based on questionnaires and not on determination of biological parameters [12, 53].

In the present study, the participants were normal “fitness-seeking” young men, at least moderately trained. In this group, it is not easy to find a correlation between VO_2max_ and the degree of either “resting” or exercise-induced OS. VO_2max_ varies over the range of 30 to ca 60 mL/min/kg, whereas no significant correlation has been observed between physical fitness and the exercise-induced change of OS, as evaluated on the basis of almost all the studied parameters (only the level of GST increased slightly with the physical fitness, not shown). This results accord with the findings of both Falone et al. [17] and Elosua et al. [12] that the physical exercise-induced change in OS, as measured by most of the parameters in sedentary subjects, is not significantly different from the change observed in trained people. Furthermore, our results are not inconsistent with those of Traustadóttir et al. [52], who found that the OS, as evaluated by several biomarkers, increased upon intense activity in sedentary subjects more than in physically fit subjects. None of our participants is sedentary.

## 4. Conclusions

Intense physical activity of young, fit, and healthy subjects is accompanied by a slight but significant, fitness-independent increase of the concentrations of lipid peroxidation products (TBARS and hydroperoxides that absorb light at 245 nm), whereas the level of oxidizable lipids (essentially polyunsaturated fatty acids) in their blood does not become more susceptible to* ex vivo* peroxidation. This conclusion is not inconsistent with those of previous studies, in which the participants where less homogeneous with respect to gender, age, and fitness and/or to the test used to evaluate the exercise-induced effects (cycling in our study, swimming, bowing, running, or ball games in most other relevant studies). Analysis of the factors responsible for the differences between the different investigations requires more data on studies that differ from one another in merely one detail. For example, to study the effect of the method of evaluation of the OS, we need data about the results of at least two experiments conducted under conditions of equal profile of the participants, equal test of fitness, and equal protocol of blood withdrawal and treatment.

The activity of antioxidant enzymes apparently increased due to physical exercise-induced OS but the increase did not reach statistical significance during the physical exercise and only after 1 hour the central enzyme CAT was found to be statistically significant higher than prior to the workout. The activity of all other studied enzymes exhibited a considerable tendency of decrease during the one-hour rest after the end of intense physical workout (recovery) to values similar to those observed before workout. These results demonstrate the efficiency of the mechanisms responsible for homeostasis of oxidative status. Given the previous observations of large differences between active and sedentary people, the observed lack of association between fitness and OS, evaluated by any of the studied methods, is consistent with the bell shaped Hormesis curve [22, 32], attributed to the need of the body for suitable ROS levels. Our interpretation of this study is that it indicates that, in young healthy men, moderate training is sufficient to keep the OS from being elevated to dangerous level upon exercise.

## Supplementary Material

Exercise-induced changes of the levels of OS biomarkers, dependencies on pre-exercise levels.

## Figures and Tables

**Table 1:** Subjects characteristics.

Parameter	Average (range)
Age (years)	26.8 (23.6; 30.3)
Height (cm)	179.1 (169.5; 193)
Weight (Kg)	77.9 (61.5; 88.5)
BMI (Kg/m^2^)	23.4 (19.2; 30.8)
Fat percentage (%)	14.0 (7.0; 27.6)
Physical activity per week (hours)	6.53 (0; 18)
VO_2max_ (mL/min/Kg)	48.9 (30.4; 62.8)
RER	1.27 (1.12; 1.56)
Rest heart rate (pulse/min)	61.6 (35; 83)
Maximum heart rate (pulse/min)	184.4 (165; 199)

**Table 2:** Average values of the OS biomarkers.

Biomarker	Rest	Workout	Recovery
	Average (range)	Average (range)	Average (range)
CAT (*μ*mol H_2_O_2_/min/*μ*g Hb)	376 (104; 783.0)	413 (137; 941)	435 (265; 808)
GPx (*μ*mol/min/g Hb)	15.7 (6.4; 26.1)	16.1 (8.0; 28.9)	16.0 (8.9; 25.8)
GST (*μ*mol/min/g Hb)	1.37 (0.62; 2.52)	1.46 (0.36; 2.62)	1.42 (0.27; 3.28)
GR (*μ*mol/min/g Hb)	3.81 (1.51; 5.48)	4.18 (1.75; 9.32)	3.91 (1.85; 6.90)
G6PD (*μ*mol/min/g Hb)	5.37 (3.24; 8.01)	5.04 (3.81; 7.39)	5.03 (3.51; 6.84)
TBARS (*μ*M)	8.84 (3.67; 26.18)	10.78 (4.67; 31.79)	8.87 (3.28; 24.80)
Lag 245 (min)	50.4 (38.4; 62.1)	49.3 (38.6; 64.0)	53.0 (42.1; 65.1)
*V*_max⁡_ 245 · 10^3^ (OD/min)	3.27 (2.44; 4.82)	3.40 (2.46; 5.10)	3.26*E* − 3 (2.44; 4.35)
OD245 (OD)	0.364 (0.277; 0.522)	0.385 (0.277; 0.565)	0.353 (0.269; 0.498)
OD245_in_ (OD)	1.15 (1.01; 1.80)	1.26 (1.09; 1.52)	1.17 (1.01; 1.55)

## Abbreviations and Symbols

AOPP: Advanced Oxidation Protein Products
CAT: Catalase
G6PD: Glucose-6-Phosphate Dehydrogenase
GPx: Glutathione Peroxidase
GR: Glutathione Reductase
GST: Glutathione S-Transferase
Lag: Time interval preceding fast peroxidation of serum lipids
LMWA: Low molecular weight antioxidants
MDA: Malondialdehyde
NOx: Nitrate/nitrite
OD_max_: Maximal optical density of peroxidized serum at 245 nm
OD245_in_: Initial optical density of serum at 245 nm
OS: Oxidative stress
PC: Protein Carbonyls
PUFA: Polyunsaturated fatty acids
RER: Respiratory exchange ratio
ROS: Reactive oxygen species
SOD: Superoxide Dismutase
TAC: Total Antioxidant Capacity
TBARS: Thiobarbituric acid reactive substances
TEAC: Trolox-Equivalent Antioxidant Capacity
*V*_max_: Maximal rate of copper induced peroxidation of serum lipids.
