# Supplementary material for: In Healthy Young Men, a Short Exhaustive Exercise Alters the Oxidative Stress Only Slightly, Independent of the Actual Fitness
Source: Oxid Med Cell Longev. 2016 Feb 17;2016:9107210. doi: 10.1155/2016/9107210 (PMC4773557; doi:10.1155/2016/9107210)

Fig. 1S. Apparent statistically significant (P<0.05) inverse correlations between physical exercise-induced changes in the levels of OS biomarkers and their initial levels (before the exercise).

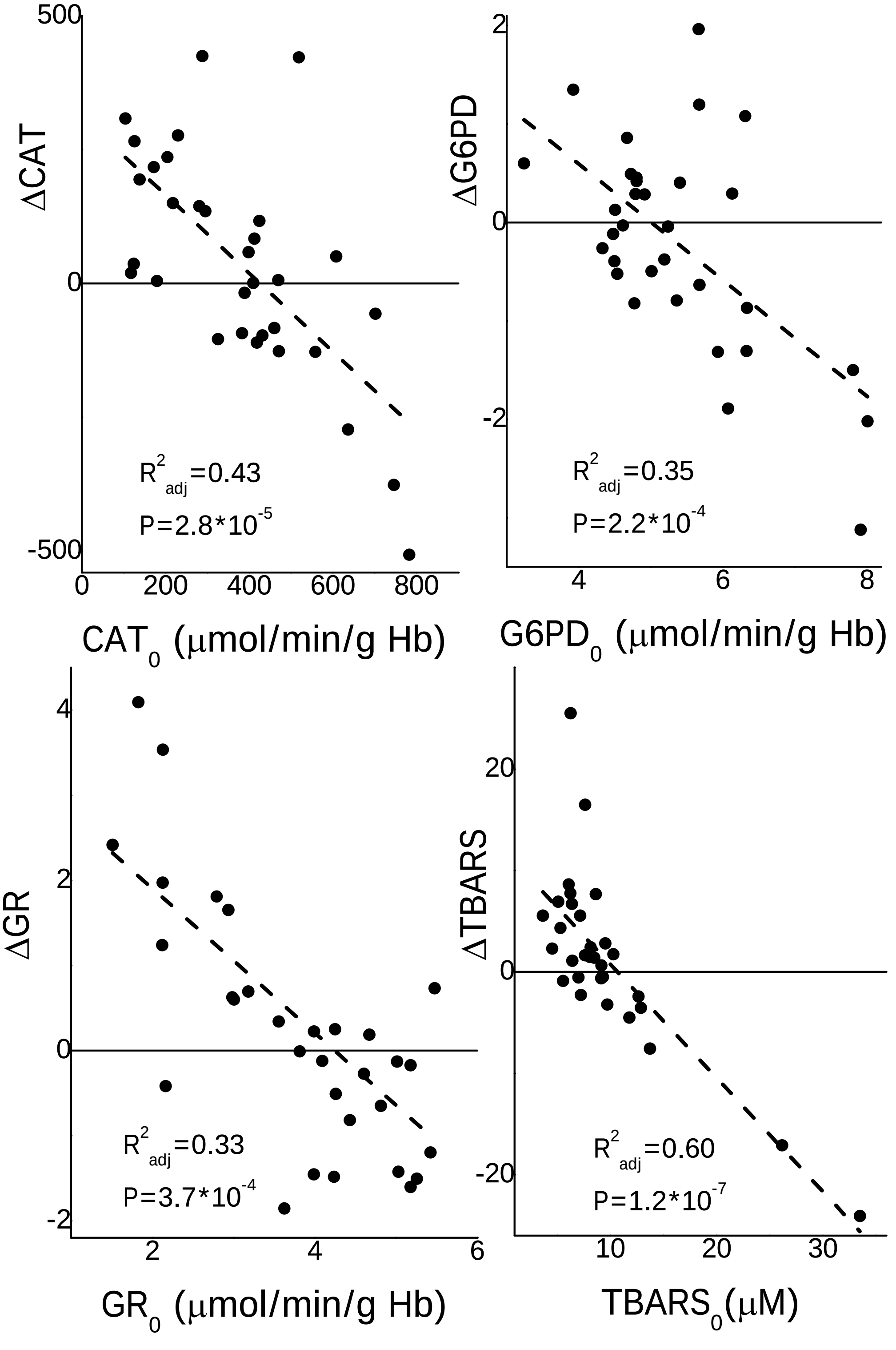

Supplement: Supplementary file 1 — Exercise-induced changes of the levels of OS biomarkers, dependencies on pre-exercise levels. [file 9107210.f1.pdf]
